# Supplementary figures and images for: Socio-spatial disparities in access to emergency health care—A Scandinavian case study
Source: PLoS One. 2021 Dec 10;16(12):e0261319. doi: 10.1371/journal.pone.0261319 (PMC8664193; doi:10.1371/journal.pone.0261319)

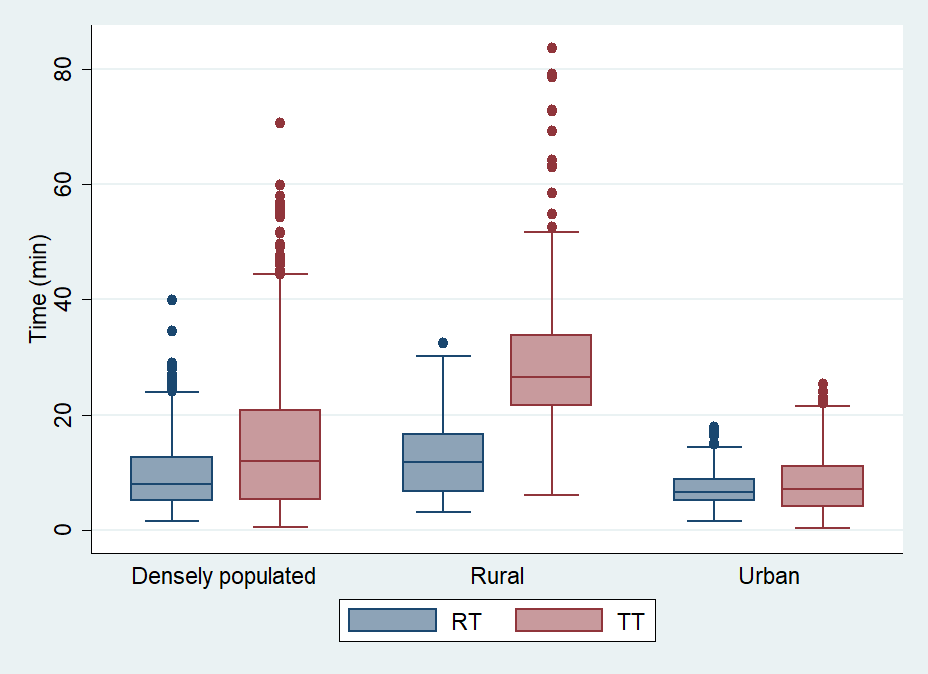

Supplement: S1 Fig — Source: Authors. (TIF) [file pone.0261319.s001.tif]
