# Supplementary material for: Socio-spatial disparities in access to emergency health care—A Scandinavian case study
Source: PLoS One. 2021 Dec 10;16(12):e0261319. doi: 10.1371/journal.pone.0261319 (PMC8664193; doi:10.1371/journal.pone.0261319)
Supplement: S1 Table — (PDF) [file pone.0261319.s002.pdf]

Type of data		Description	Year	Source	Type
Spatial divisions	NUTS	A system of spatial division of areas and regions in the EU.	2020	European Union (Eurostat)	Shapefile (polygon)
	Regions	Administrative borders for regions.	2018	Lantmäteriet	Shapefile (polygon)
	Municipalities	Administrative borders for municipalities.	2018	Lantmäteriet	Shapefile (polygon)
	DeSO	Statistical areas with around 1000-2000 persons in each area.	2018	Statistics Sweden	Shapefile (polygon)
Road network and facilites	NVDB	A national database containing all roads in Sweden.	2020	The Swedish Transportation Agency	Shapefile (line)
	Ambulance stations	Ambulance stations addresses obtained from the individual regions	2020	Administrative regions	Shapefile (point)
	Hospitals	Hospitals with emergency rooms.	2020	The Swedish National Board of Health and Welfare	Shapefile (point)
Population data	Population numbers	Grid with 1x1 km^2 squares containing total population numbers.	2018	Statistics Sweden	Shapefile (square)
Socioeconomic and demographic variables	Age	Share of population aged 64 and above per DeSO-area	2018	Statistics Sweden	Share
	Income	Median income levels per DeSO-area	2018	Statistics Sweden	Cont.
	Education	Share of population with 3 or more years of tertiary education per DeSO-area	2018	Statistics Sweden	Share
