# Supplementary material for: Socio-spatial disparities in access to emergency health care—A Scandinavian case study
Source: PLoS One. 2021 Dec 10;16(12):e0261319. doi: 10.1371/journal.pone.0261319 (PMC8664193; doi:10.1371/journal.pone.0261319)
Supplement: S2 Table — * p<0.05. (PDF) [file pone.0261319.s003.pdf]

	1.	2.	3.	4.	5.	6.	7.
1. Older adults	1.0000						
2. High education	-0.2506*<br>0.0000	1.0000					
3. Median income	-0.0223<br>0.2621	0.4972*<br>0.0000	1.0000				
4. Below median income	0.0029<br>0.8828	-0.2221*<br>0.0000	-0.6793*<br>0.0000	1.0000			
5.Unemployed	-0.2387*<br>0.0000	-0.1762*<br>0.0000	-0.8526*<br>0.0000	0.6555*<br>0.0000	1.0000		
6. Foreign born	-0.4023*<br>0.0000	-0.1963*<br>0.0000	-0.6697*<br>0.0000	0.5429*<br>0.0000	0.8337*<br>0.0000	1.0000	
7. Ambulance station	0.0279<br>0.1614	0.0300<br>0.1326	-0.0855*<br>0.0000	0.0760*<br>0.0001	0.0676*<br>0.0007	0.0453*<br>0.0229	1.0000
