# Supplementary material for: Socio-spatial disparities in access to emergency health care—A Scandinavian case study
Source: PLoS One. 2021 Dec 10;16(12):e0261319. doi: 10.1371/journal.pone.0261319 (PMC8664193; doi:10.1371/journal.pone.0261319)
Supplement: S3 Table — *All values in minutes. (PDF) [file pone.0261319.s004.pdf]

		Urban			Densely populated			Rural			Total		
**Region**	**Stat.**	**RT**	**TT**	**TPT**	**RT**	**TT**	**TPT**	**RT**	**TT**	**TPT**	**RT**	**TT**	**TPT**
Blekinge	Mean	-	-	-	12.1	20.5	46.1	10.0	23.0	48.1	11.5	21.2	46.7
	Median	-	-	-	10.9	18.5	51.0	10.8	22.5	47.1	10.9	21.6	48.2
	90th centile	-	-	-	23.4	37.0	70.9	15.1	27.8	58.5	22.3	36.6	66.2
	N.	-	-	-	67	67	67	26	26	26	93	93	93
Halland	Mean	-	-	-	9.2	15.3	38.0	11.3	27.2	53.7	9.5	16.8	39.9
	Median	-	-	-	7.7	15.3	40.8	11.6	23.2	53.4	8.1	17.9	41.7
	90th centile	-	-	-	16.8	26.6	53.9	20.6	40.3	69.4	16.9	29.9	57.2
	N.	-	-	-	163	163	163	23	23	23	186	186	186
Jönköping	Mean	-	-	-	9.6	16.0	39.0	12.9	29.0	56.9	10.3	18.7	42.8
	Median	-	-	-	8.3	12.4	36.0	13.3	30.1	54.5	8.8	17.8	40.8
	90th centile	-	-	-	16.6	37.1	59.2	22.6	35.4	72.2	18.7	35.4	63.3
	N.	-	-	-	167	167	167	44	44	44	211	211	211
Kalmar	Mean	-	-	-	10.1	15.4	39.0	11.4	33.1	59.5	10.5	21.0	45.5
	Median	-	-	-	8.6	9.7	34.4	11.3	29.9	56.3	9.0	19.3	44.9
	90th centile	-	-	-	19.9	42.0	68.2	20.4	48.3	77.1	20.4	44.9	69.6
	N.	-	-	-	109	109	109	50	50	50	159	159	159
Kronoberg	Mean	-	-	-	9.7	12.2	35.4	13.4	30.7	59.2	11.0	18.6	43.7
	Median	-	-	-	7.6	7.3	29.4	11.6	30.3	55.8	8.8	17.4	42.8
	90th centile	-	-	-	19.8	32.0	55.8	25.3	44.1	83.4	21.5	35.8	69.9
	N.	-	-	-	73	73	73	39	39	39	112	112	112
Skåne	Mean	8.0	6.7	28.2	9.2	14.1	36.8	12.0	24.4	51.6	9.2	12.7	35.6
	Median	8.0	5.1	26.8	8.4	13.7	35.7	12.6	23.5	50.6	8.4	10.4	32.8
	90th centile	11.2	13.2	36.6	16.4	26.0	54.1	18.8	33.6	65.6	15.3	26.1	54.3
	N.	318	318	318	348	348	348	120	120	120	786	786	786
Västra Götaland	Mean	6.2	9.0	28.6	8.6	13.4	35.5	12.5	30.3	57.9	8.4	15.0	37.2
	Median	5.6	8.5	27.5	7.0	9.9	32.7	11.8	27.5	54.5	6.4	11.1	32.5
	90th centile	10.2	15.4	38.1	16.5	27.7	54.6	21.2	45.8	76.5	16.6	31.6	62.6
	N.	389	389	389	408	408	408	196	196	196	993	993	993
Total	Mean	7.0	7.9	28.4	9.3	14.6	37.4	12.2	28.6	55.9	9.2	15.5	38.5
	Median	6.6	7.1	27.3	8.0	11.9	35.2	11.8	26.4	53.3	7.9	12.2	35.1
	90th centile	10.8	14.4	37.2	17.2	30.6	57.3	20.4	42.1	72.2	17.0	32.5	60.8
	Min	1.6	.3	18.8	1.5	.4	15.7	3.2	6	30.8	1.5	0.3	15.7
	Max	17.8	25.4	52.7	39.8	70.6	100.7	32.4	83.6	108	39.8	70.6	108
	N.	707	707	707	1335	1335	1335	498	498	498	2540	2540	2540
